# Antimicrobial Peptides with Enhanced Salt Resistance and Antiendotoxin Properties

**DOI:** 10.3390/ijms21186810

**Published:** 2020-09-16

**Authors:** Hung-Lun Chu, Ya-Han Chih, Kuang-Li Peng, Chih-Lung Wu, Hui-Yuan Yu, Doris Cheng, Yu-Ting Chou, Jya-Wei Cheng

**Affiliations:** 1 Institute of Biotechnology and Department of Medical Science, National Tsing Hua University, Hsinchu 300, Taiwan; edchu09@gmail.com (H.-L.C.); eva.chih@risebiopharma.com (Y.-H.C.); richard850210@gmail.com (K.-L.P.); ericwu.cl@gmail.com (C.-L.W.); gwalt1103@gmail.com (H.-Y.Y.); ytchou@life.nthu.edu.tw (Y.-T.C.); 2 Department of Radiology, Harbor-UCLA Medical Center, 1000 West Carson Street, Torrance, CA 90509, USA; dorisc5558@gmail.com

**Keywords:** antimicrobial peptide, salt resistance, lipopolysaccharide, antiendotoxin, cecropin-like

## Abstract

A strategy was described to design antimicrobial peptides (AMPs) with enhanced salt resistance and antiendotoxin activities by linking two helical AMPs with the Ala-Gly-Pro (AGP) hinge. Among the designed peptides, KR12AGPWR6 demonstrated the best antimicrobial activities even in high salt conditions (NaCl ~300 mM) and possessed the strongest antiendotoxin activities. These activities may be related to hydrophobicity, membrane-permeability, and α-helical content of the peptide. Amino acids of the C-terminal helices were found to affect the peptide-induced permeabilization of LUVs, the α-helicity of the designed peptides under various LUVs, and the LPS aggregation and size alternation. A possible model was proposed to explain the mechanism of LPS neutralization by the designed peptides. These findings could provide a new approach for designing AMPs with enhanced salt resistance and antiendotoxin activities for potential therapeutic applications.

## 1. Introduction

Antimicrobial peptides (AMPs) have been found in the innate defense systems of plants, insects, and animals [1,2,3,4,5]. AMPs can incorporate and disturb microbial membranes and hence cause their death [6,7]. The modes of action of AMPs have been widely described to include the barrel-stave model, the toroidal pores model, and the carpet model [5,8]. Moreover, AMPs can act synergistically with current antibiotics to reduce bacterial resistance and reduce the amount of antibiotics needed [9,10,11]. Owing to these unique mechanisms, AMPs may be the solution to the problem of bacterial resistance [12,13].

Problems such as salt sensitivity, cost of synthesis, bioavailability, and stability have limited the therapeutic applications of antimicrobial peptides [14,15]. Among these problems, salt sensitivity is directly related to the microbicidal mechanism of antimicrobial peptides. For example, the efficacy of the clinically active peptide P-113 is greatly reduced in high salt conditions [15]. Similar problems have also been found with other antimicrobial peptides [16,17,18].

Lipopolysaccharide (LPS, endotoxin) is the major outer surface membrane component of Gram-negative bacteria [19]. LPS forms an amphiphilic structure that consists of three regions: a conserved lipid A motif, a highly variable polysaccharide or O antigen, and a core oligosaccharide. LPS released from bacteria into the bloodstream during infection may interact with Toll-like receptor 4 (TLP4) on macrophages and subsequently activate the transcription factor nuclear factor-kappa B (NF-kB) to cause serious unwanted stimulation of the host’s immune system and lead to septic shock of the patient [20,21]. Neutralization of LPS using anti-LPS or anti-TNF-α antibodies had only limited success in the treatment of sepsis [22,23]. Various studies have shed light on AMPs with antiendotoxin properties [24,25,26,27,28,29,30,31,32,33,34,35]. Some AMPs have been shown to bind to LPS and neutralize LPS stimulated proinflammatory responses [26,29,36]. However, rules governing the design of AMPs with antiendotoxin properties are still not very clear [26,29,36].

Recently, solution structures of S1 (Ac-KKWRKWLAKK-NH_2_) and S1-Nal-Nal (Ac-KKWRKWLAKKNal-Nal-NH_2_) in complex with LPS micelles have been reported [37]. Both S1 and S1-Nal-Nal bound to LPS through the hydrophilic surface of their helices to the negatively charged region of LPS. S1-Nal-Nal further inserted deeper into the hydrophobic core of LPS micelles and created better hydrophobic interactions of its C-terminal β-naphthylalanine end-tags with the lipid A motif of LPS. The LPS-induced inflammation might then be prohibited. Moreover, a strategy was developed to increase salt resistance and LPS neutralization activities of P-113 (AKRHHGYKRKFH-NH_2_) by replacing histidines with phenylalanine- (Phe-P-113), β-naphthylalanine- (Nal-P-113), β-diphenylalanine- (Dip-P-113), and β-(4,4′-biphenyl)alanine- (Bip-P-113). Structure−activity relationships of P-113 and its derivatives were evaluated [38]. Among these peptides, Bip-P-113 with the longest bulky non-nature amino acid sidechains was discovered to possess enhanced salt resistance, serum proteolytic stability, peptide-induced permeabilization, zeta potentials, LPS aggregation, and in vitro and in vivo LPS neutralizing activities.

Cecropins are a family of antimicrobial peptides widely found in the innate immune system of Cecropia moth. Cecropins exhibit broad spectra antimicrobial and anticancer activities [39,40,41]. The structures of cecropins are composed of 34−39 amino acids with an N-terminal amphipathic α-helix, an AGP hinge and a hydrophobic C-terminal α-helix. Recently, cecropin A and the cecropin-like peptide papiliocin were found to possess anti-inflammatory activities in LPS-stimulated murine macrophage [42,43]. Studies of cecropin analogues [44], cecropin A/cecropin B hybrids [45], and cecropin A, LL-37, and magainin hybrids also revealed potential antimicrobial and anticancer activities [46].

We hypothesize that the binding and neutralization of LPS of cecropin and cecropin-like peptides is through similar structural features like S1-Nal-Nal (i.e., amphipathic helix−linker−hydrophobic terminus). Here, we use this structural feature to create a new type of antimicrobial peptides by linking an amphipathic peptide and a hydrophobic peptide with the AGP sequence. The antimicrobial and LPS neutralization activities of these designed peptides were determined.

## 2. Results

### 2.1. Antimicrobial Peptides

**Figure 1:**
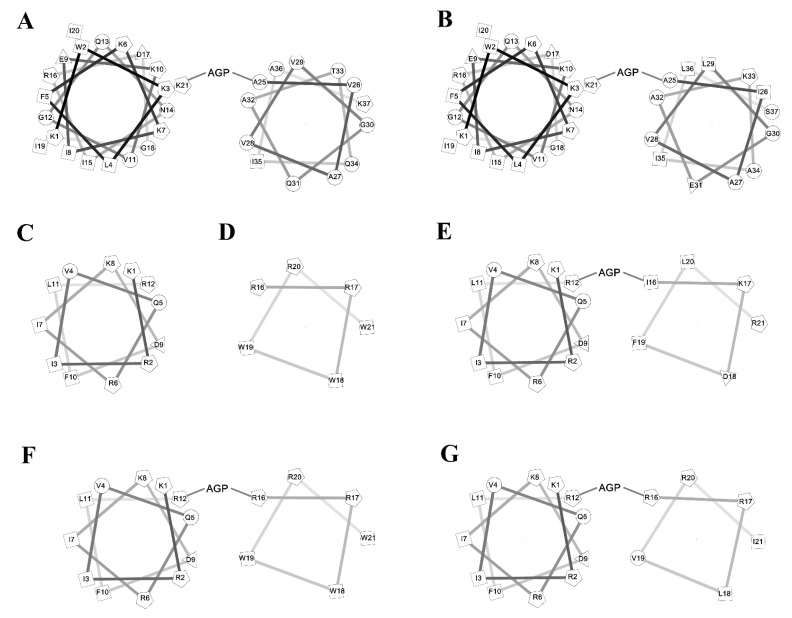
Helical wheel of the peptides. (**A**) cecropin A, (**B**) cecropin B, (**C**) KR12, (**D**) RW6, (**E**) KR12AGPKR6, (**F**) KR12AGPWR6, (**G**) KR12AGPVR6.

To investigate the amphipathic helix–AGP–hydrophobic helix on the antimicrobial and neutralization activities of LPS, two peptides KR12 and RW6 were used for the N-terminal and C-terminal helices. KR-12 (KRIVQRIKDFLR) was derived from human host defense cathelicidin LL-37 and was found to possess antimicrobial activities against Gram-negative and Gram-positive bacteria. RW6 (RRWWRW) was derived from the reversed sequence of WR6 (WRWWRR) which had moderate antimicrobial activities [47,48,49,50]. Sequences of the designed peptides are listed in Table 1 and their helical wheel analyses are shown in Figure 1. KR12AGPKR6 was named based on the “KR12–AGP–KR6 sequence”. KR12AGPWR6 was named based on the “KR12–AGP–WR6 sequence”. KR12AGPVR6 were named based on the “KR12–AGP–VR6 sequence”. KR12AGPKR6, KR12AGPWR6 and KR12AGPVR6 were designed to compare the effects of hydrophobicity, membrane-permeability, and α-helical contents. All peptides were acetylated and amidated at the N- and C-terminus.

### 2.2. Antibacterial Activity and Salt Resistance

**Figure 2:**
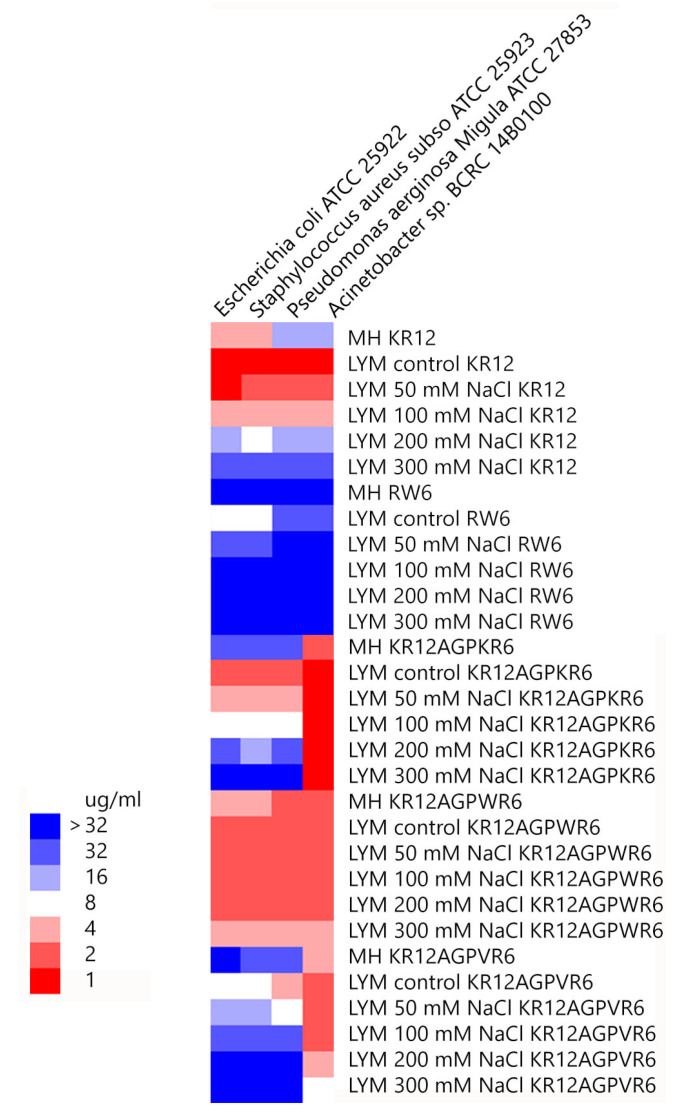
Minimal inhibitory concentration (MIC) values were displayed on a color scale for KR12, RW6, KR12AGPKR6, KR12AGPWR6 and KR12AGPVR6 under Mueller−Hinton (MH) broth and LYM medium with different concentrations of NaCl.

Minimal inhibitory concentration values (MICs) of the peptides were determined against Gram-positive and Gram-negative bacteria in Mueller−Hinton (MH) broth or LYM broth media (with 50, 100, 200 or 300 mM NaCl added) (Figure 2). KR12 was very effective against both Gram-positive and negative bacteria in LYM medium (MIC ~1 μg/mL). However, MIC values of KR12 increased to 4~16 μg/mL in MH broth and ~32 μg/mL in LYM broth with 300mM NaCl added. RW6 had only limited antimicrobial activity (8~32 μg/mL) in LYM broth and lost its antibacterial activity in MH broth and in LYM broth with >100 mM NaCl added. Similar to KR12, KR12AGPKR6 had MICs 1~2 μg/mL against both Gram-positive and negative bacteria in LYM medium. However, KR12AGPKR6 lost its antibacterial activity gradually to ~32 μg/mL in MH broth. KR12AGPKR6 was completely inactive in LYM broth with 300 mM NaCl added for *Escherichia coli*, *Staphylococcus aureus*, and *Pseudomonas aerginosa Migula*. Surprisingly, KR12AGPKR6 still possessed effective antibacterial activity against *Acinetobacter sp.* In MH broth (~2 μg/mL) and in LYM broth with 300 mM NaCl added (~1 μg/mL). On the other hand, KR12AGPWR6 displayed superior antibacterial activities against both Gram-positive and Gram-negative bacteria in MH and LYM media even in NaCl ~300 mM (MIC 2~4 μg/mL). KR12AGPVR6 exhibited moderate activities in MH broth (4~32 μg/mL), and lost its activity in LYM broth with 300 mM NaCl added. In addition, KR12, KR12AGPKR6, KR12AGPWR6 and KR12AGPVR6 were more effective to inhibit *Acinetobacter* sp. than the other three bacteria strains.

### 2.3. Limulus Amebocyte Lysate (LAL) Assay

**Figure 3:**
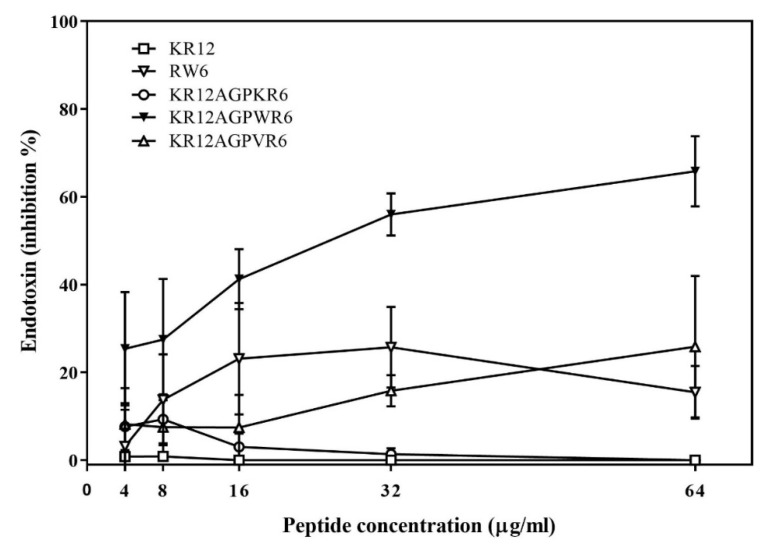
Lipopolysaccharide (LPS) -neutralizing activities determined by *limulus* amebocyte lysate (LAL) assay. The experiments were performed in triplicate. Results are presented as means ± standard deviations (SD).

The ability of binding and neutralizing LPS in vitro was measured by the LAL assay which is well-known as the most sensitive and specific method [29,33,49,51]. As shown in Figure 3, KR12AGPWR6 blocked the interaction between LPS and factor C (an LPS sensitive serine protease isolated from the hemocyte granules of the horseshoe crab Limulus), and neutralized the downstream reaction dose-dependently (greater than 60% inhibition at 64 μg/mL). KR12, RW6, KR12AGPKR6 and KR12AGPVR6 had only limited effects (less than 25% inhibition at 64 μg/mL).

### 2.4. Cytotoxicity

**Figure 4:**
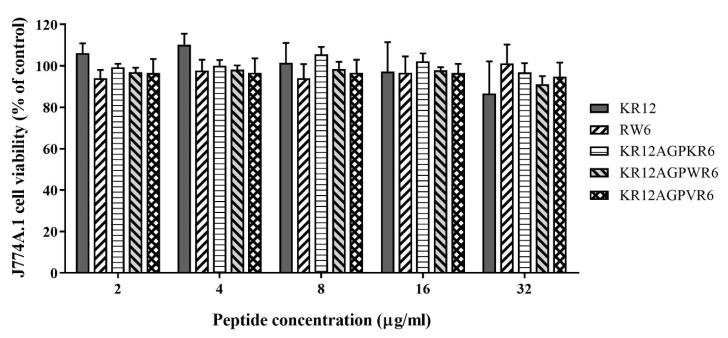
Cytotoxicity to murine macrophage J744A.1 cells of KR12, RW6, KR12AGPKR6, KR12AGPWR6 and KR12AGPVR6. Data are representative of at least three independent experiments and results are presented as means ± standard deviations (SD).

Cytotoxicity of the designed peptides was evaluated by MTT assay using murine macrophage J744A.1 cells. RW6, KR12AGPKR6, KR12AGPWR6, and KR12AGPVR6 all exhibited little or even no cell toxicity (Figure 4). However, KR12 showed greater toxicity at 64 μg/mL (cell survival rate less than 40%).

### 2.5. Inhibition of Endotoxin-Induced Inflammation

**Figure 5:**
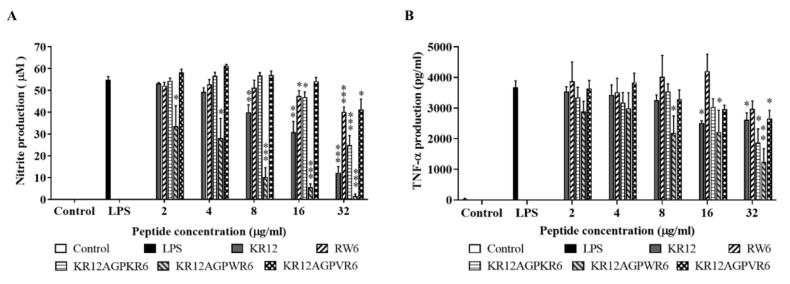
Inhibition of (**A**) LPS-induced nitric oxide (NO) production and (**B**) TNF-α release by the designed peptides with 2–32 μ-/mL working concentration in murine macrophage J774A.1 cells. Data are representative of at least three independent experiments and results are presented as means ± standard deviations. * *p* < 0.05; ** *p*  <  0.01; *** *p*  <  0.001, student’s t-test versus LPS.

LPS (lipopolysaccharide) can induce nitrite oxide production in macrophage cells [52]. Among the peptides studied, KR12AGPWR6 demonstrated the best ability to inhibit LPS-induced NO production in murine macrophage J744A.1 cells (Figure 5A).

TNF-α plays an important role in septic shock and is generally used as an indicator for septic shock [52]. Similar to the results of LPS-induced nitrite oxide production in murine macrophage J774A.1 cells above, KR12AGPWR6 showed the best ability to inhibit TNF-α release (Figure 5B).

### 2.6. Endotoxemia Mouse Model

**Figure 6:**
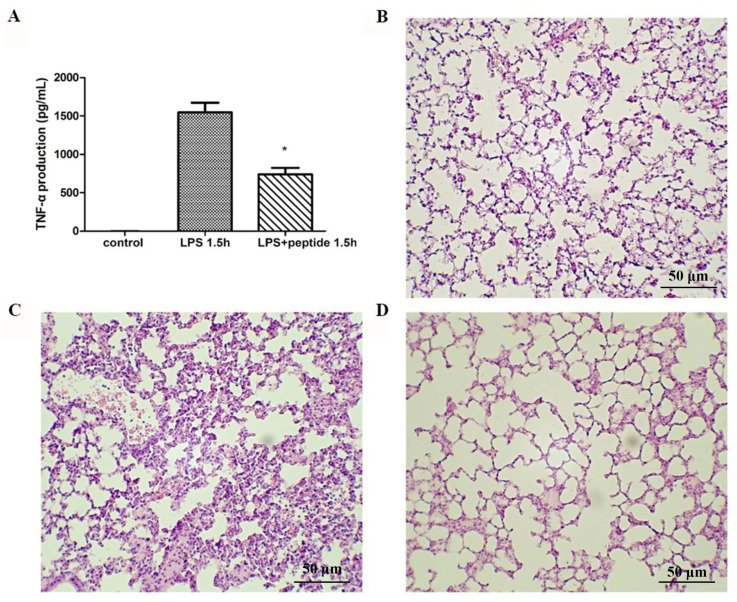
Suppression of LPS-stimulated inflammation in endotoxemic mice (C57BL/6) by KR12AGPWR6. (**A**) LPS-stimulated TNF-α release 1.5 h after injection. Excised lungs from the sacrificed mice 24 h after injection were subjected to H&E staining (**B**) control, (**C**) LPS (**D**) LPS and peptide. KR12AGPWR6 displayed the protective activity on LPS-stimulated endotoxemic mice. All scale bars = 50 μm. Results are presented as means ± standard deviations (SD); *n* = 5. * *p* < 0.05 versus LPS.

To evaluate the anti-LPS effect of KR12AGPWR6 in vivo, the mice were divided into three groups and received PBS, LPS, and a mixture of LPS and peptide, respectively [29]. Blood was collected via the tail veins of the mice 1.5 h after injection. Serum TNF-α production increased dramatically in the LPS group. The KR12AGPWR6-treated mice displayed a significantly lower level of TNF-α (Figure 6A). Pathological evaluation of the lung tissues revealed that the proliferation of alveolar epithelial cells and pulmonary hemorrhage were reduced in mice treated with KR12AGPWR6 (Figure 6B–D).

### 2.7. Peptide-Induced Permeabilization, Circular Dichroism (CD) Spectroscopy, and LPS Aggregation

We have used peptide-induced permeabilization of large unilamellar vesicles (LUVs), CD spectroscopy, and LPS aggregation studies to investigate the factors attributed to the differences of antibacterial and antiendotoxin activities among KR12AGPKR6, KR12AGPWR6 and KR12AGPVR6.

The membrane-permeabilizing abilities of peptides were investigated by releasing calcein from phospholipid vesicles with different surface charge densities. POPC/cholesterol LUVs with neutral charge were used to mimic the mammalian cell membrane. POPC/POPG LUVs with negative charge were used to mimic the anionic bacterial membrane, and POPC/LPS LUVs were serving as Gram-negative bacterial membranes which contain lipopolysaccharides.

### 2.8. Peptide-Induced Permeabilization

**Figure 7:**
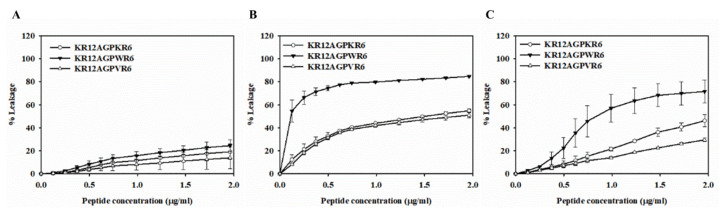
Peptide-induced permeabilization of LUVs by the designed peptides. Plot showed the percentage of calcein leakage of, KR12AGPKR6, KR12AGPWR6 and KR12AGPVR6 in 10 µM (**A**) POPC/cholesterol, (**B**) POPC/LPS and (**C**) POPC/POPG LUVs. Results are presented as means ± standard deviations (SD); *n* = 3.

When peptides lysed or disrupted lipid membrane, the entrapped calcein got released into the buffer. All three peptides displayed weak leakages on POPC/cholesterol LUVs (Figure 7A). KR12AGPKR6, KR12AGPWR6 and KR12AGPVR6 were shown to possess dose-dependent calcein leakage activities on POPC/LPS and POPC/POPG LUVs (Figure 7B,C). Among these peptides, KR12AGPWR6 demonstrated the strongest calcein leakage on POPC/LPS and POPC/POPG LUVs with about 80% and 70% leakage rate, respectively. The results of dye leakages indicated that the activities of the peptides to induce calcein release from negatively charged LUVs were concordant with their antibacterial and anti-LPS activities.

### 2.9. CD Spectroscopy

**Figure 8:**
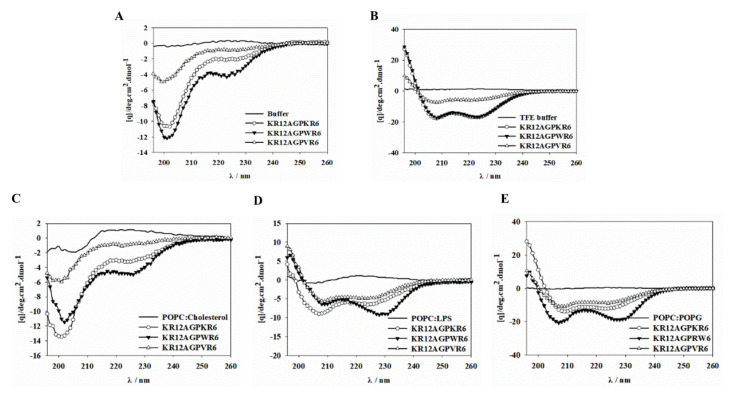
Circular dichroism spectra of the designed peptides. CD spectra were recorded at 60 µM concentration of KR12AGPKR6, KR12AGPWR6 and KR12AGPVR6 in (**A**) 20 mM phosphate buffer, (**B**) 30% TFE buffer, (**C**) 1 mM POPC/cholesterol, (**D**) 1 mM POPC/LPS and (**E**) 1 mM POPC/POPG LUVs at pH7.4, 25 °C.

The secondary structures of these designed peptides dissolved in 20 mM phosphate buffer with 30% TFE or various lipid membranes were evaluated by CD spectra. All of the three peptides showed random or weak structure in aqueous solution and POPC/cholesterol LUVs (Figure 8A,C). However, these three peptides formed α-helical structures in TFE, POPC/LPS and POPC/POPG environments (Figure 8B,D,E). The degree of helicity in the POPC/LPS and POPC/POPG environments was found to be KR12AGPWR6 > KR12AGPKR6 > KR12AGPVR6. This data demonstrated that helicity of the peptides in negatively charged model membranes were inconsistent with their antibacterial and anti-LPS activities.

### 2.10. LPS Aggregation

**Figure 9:**
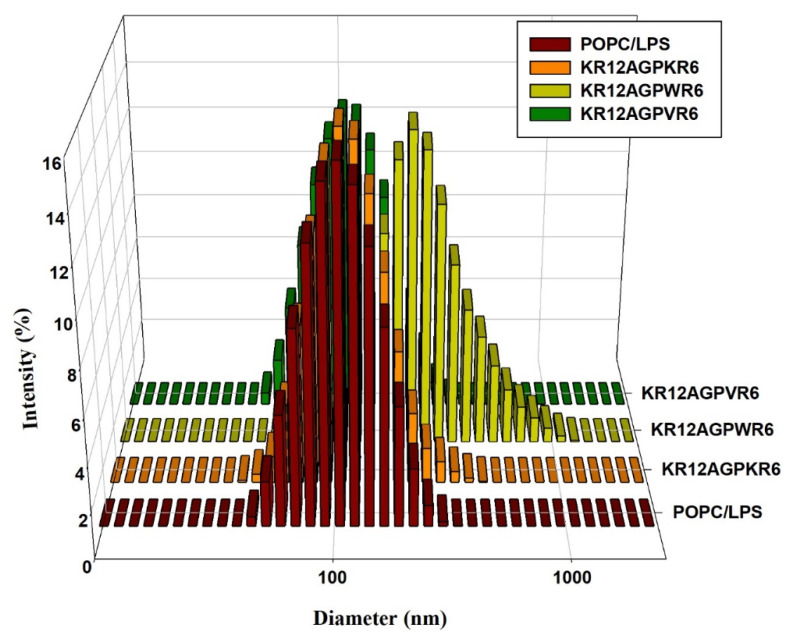
Size distribution of LPS aggregates in the presence of the designed peptides. Plots showed the size alterations of LPS aggregates in the absence or presence of KR12AGPKR6, KR12AGPWR6 and KR12AGPVR6 in 25 μM POPC/LPS solutions.

It has been reported that LPS aggregation promoted by polymyxin B and rBPI_21_ might inhibit the interaction of LPS with its cell receptors and hence block cytokine production [27,53]. Dynamic light scattering (DLS) was used to measure the size increase of the designed peptides [27,53]. The results indicated that KR12AGPWR6 promoted POPC/LPS LUVs aggregation and increased their mean sizes. KR12AGPKR6 and KR12AGPVR6 did not have the abilities in aggregating and increasing particle size (Figure 9).

## 3. Discussion

It has been shown that hydrophobicity is a key factor in the development of salt resistant and LPS-neutralizing AMPs [25,36,54]. For example, the effect of the hydrophobicity to net positive charge ratio on antibacterial and antiendotoxin activities has been reported [55]. Studies of NK-2 and N-acylated lactoferricin-derived LF11 also demonstrated that a hydrophobic interaction would increase LPS neutralization significantly for AMPs [36]. Recently, a strategy to increase salt resistance and LPS neutralization of short AMPs was developed by adding β-naphthylalanine end-tags to their termini [15,16,55]. The addition of fatty acid, vitamin E, or cholesterol to the termini of AMPs was shown to have similar results [25,56,57,58,59,60]. Along with the present studies, it is suggested that modulating the lipophilicity of the termini is very important in the design of AMPs with improved salt resistance and LPS neutralization effects.

It was shown that the solution structure of the Trp-rich antimicrobial peptide PEM-2-W5K/A9W could inset more deeply into the DPC micelles and possessed a larger buried hydrophobic surface than its parent peptide PEM-2 [61]. Results from fluorescence quenching and dye leakage experiments also showed a direct relationship between membrane-bound hydrophobic surface area and the salt-resistance of antimicrobial peptides [61]. Herein, we have calculated the hydrophobicity of the N-terminal amphipathic helix and the hydrophobicity of the C-terminal helix of each designed peptide (Table 1). Surprisingly, the hydrophobicity of the C-terminal helices of KR12AGPKR6, KR12AGPWR6 and KR12AGPVR6 correlated well with the antimicrobial and salt resistant activities as well as the in vitro and in vivo LPS-neutralizing activities. Moreover, results of the peptide-induced permeabilization of LUVs, α-helicity of the designed peptides under various LUVs, and LPS aggregation and size alternation also correlated with the calculated hydrophobicity of the C-terminal helices.

In addition to cecropin-like AMPs, many other anti-LPS AMPs may adopt similar structural features. For example, the chicken cathelicidin fowlicidin-2, MSI-594, SMAP-29, pardaxin, cecropin A, and papiliocin all have an N-terminal amphipathic helix, a central hinge, and a C-terminal hydrophobic helix [34,43,62,63,64,65]. The reversed structural features (i.e., hydrophobic helix–hinge–amphipathic helix) are also found in the chicken cathelicidins fowlicidin-1 and fowlicidin-3 [66].

**Figure 10:**
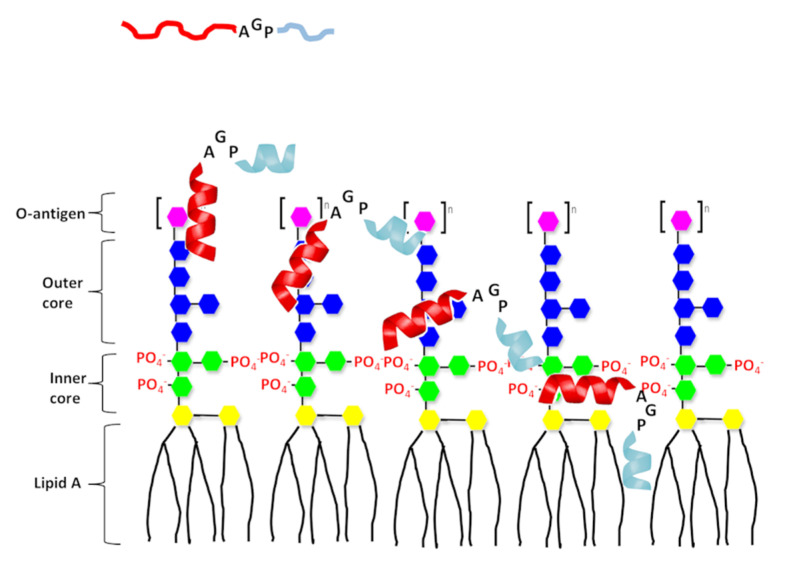
A proposed model of interaction of the designed peptide with LPS. The N-terminal helix of designed peptides shown in red and C-terminal hydrophobic helix shown in light blue. Structure of LPS consists three regions: polysaccharide O antigen (purple), core oligosaccharide (blue presents outer core and green presents inner core) and lipid A region. Lipid A consists of two glucosamine (yellow) units with attached acyl chains.

Based on our present results and the above-mentioned anti-LPS AMPs, we propose a possible model to explain the mechanism of the helix−hinge−helix peptides in the interaction with LPS (Figure 10). Firstly, the peptide is attracted to LPS by the electrostatic interactions between the N-terminal amphipathic helix and the negatively charged region of LPS. Then the C-terminal hydrophobic helix inserts itself into LPS by hydrophobic interactions with the lipid A region of LPS. The LPS-induced inflammation is then prohibited by the blocked lipid A region and aggregated LPS vesicles.

In summary, we describe a strategy to design AMPs with enhanced salt resistance and antiendotoxin activities by linking two helical AMPs with the AGP hinge. Among the designed peptides, KR12AGPWR6 demonstrated the best antibacterial activities even in high salt conditions (NaCl ~300 mM) and possessed the strongest antiendotoxin activities. These activities may be related to hydrophobicity, membrane-permeability, and α-helical content of the peptide. Our results provide a new approach to design and development of AMPs with antimicrobial and antiendotoxin activities for potential therapeutic applications.

## 4. Methods

All peptides were purchased from Kelowna Int’l Scientific Inc. (Taipei, Taiwan). The identity of the peptides was checked by matrix-assisted laser desorption-ionization/time-of-flight (MALDI-TOF) Autoflex III mass spectroscopy (Bruker Daltonik GmbH, Bremen, Germany)) and the purity (>95%) was assessed by Waters 2796 BioSeparations Module HPLC (Waters, Milford, MA, USA). POPC and POPG were purchased from Avanti Polar Lipids, Inc. (Alabaster, AL, USA). Lipopolysaccharides from *Escherichia coli* O26:B6, cholesterol and calcein were purchased from Sigma Aldrich (St. Louis, MO, USA).

### 4.1. Bacteria Culture

*Escherichia coli* strain (ATCC 25922), *Staphylococcus aureus* sp. strain (ATCC 25923), *Pseudomonas aeruginosa* Migula strain (ATCC 27853) and *Acinetobacter* sp. (BCRC number 14B0100) were used to test the antibacterial activity of the peptides. All bacteria were purchased from Food Industry Research and Development Institute (Hsinchu, Taiwan), and *Acinetobacter* sp. is multiresistant to Ampicillin, Cefazolin, Cefoxitin, Ceftazidime, Ceftriaxone, Ciprofloxacin, Penicillin, Tetracycline and Imipenem. Bacteria were cultured in sterilized MH (Müller−Hinton) broth at 200 rpm and 37 °C for 8 h. After 8 h of culture, the concentrations of the inoculums were determined by measuring absorbance of optical density at 600 nm (OD 600 = 1, equal to approximately 10^8^ CFU/mL) with Ultrospec 2100 pro UV−Visible spectrophotometer (Biochrom Ltd., Cambridge, UK)).

### 4.2. Antimicrobial Activity

The antibacterial activities were determined by the standard broth microdilution method of the National Committee for Clinical Laboratory Standards with the MH and LYM broth. The LYM broth contains 5.4 mM KCl, 5.6 mM Na_2_HPO_4_, 0.5 mM MgSO_4_, and 1.0 mM sodium citrate. In addition, 0.4 mg of ZnCl_2_, 2.0 mg of FeCl_3_·6H_2_O, 0.1 mg of CuSO_4_·5H_2_O, 0.1 mg of MnSO_4_·H_2_O, 0.1 mg of Na_2_B_4_O_7_·10H_2_O, 700 mg of amino acid mixtures without tryptophan (Clontech), and 20 mg of L-tryptophan were added per liter of medium. A vitamin mixture (100X, Sigma) and glucose at final concentration of 2% were also added. We made 1 µL peptide solutions (ranging from 3200 µg/mL to 100 µg/mL in serial dilution) and mixed with 99 µL inoculum (5 × 10^5^ CFU/mL) in a polypropylene 96-well plate. We measured the turbidity at OD 600 nm by ELISA plate reader (Thermo Max, Molecular Devices, Sunnyvale, CA, USA). The absorbance of culture medium and inoculum suspension without peptides were used as the negative and positive control, respectively. The MIC value is the lowest concentration of peptide at which there is no obvious growth (equal or more than 90%). MICs were converted to a color scale and displayed using the TreeView Program [57,67]. All peptides were tested in triplicate.

### 4.3. Binding and Neutralization of Peptides to LPS

The abilities of designed peptides to bind and neutralize LPS were assessed using *Limulus* amebocyte lysate (LAL) assay (Cape Cod Inc., East Falmouth, MA, USA). LAL is an extract of amebocytes from the Atlantic horseshoe crab *Limulus Polyphemus*. LAL reacts with lipopolysaccharide (LPS) from bacteria. Different concentrations (5, 4, 2, 1, 0.5 EU) of control standard LPS (CSE) were mixed with LAL reagent water at the same volume of 25 μL to make a standard curve. The samples of 25 μL of different peptide concentrations (128, 64, 32, 16, 8 μg/mL) were mixed with 25 μL CSE (5 EU) in a 96-well plate. A portion of 50 μL of pyrochrome reagent was added to the wells immediately. The absorbance at 405 nm was measured by microplate reader at 37 °C every minute until 25 min.

### 4.4. Cell Culture

The murine macrophage cell line J774A.1 was received from Dr. Wen-Ching Wang, Institute of Molecular & Cellular Biology, National Tsing Hua University [68]. Cells were cultured in DMEM (Dulbecco’s modified minimal essential medium) medium supplemented with 10% bovine calf serum and antibiotic at 37 °C in 5% CO_2_.

### 4.5. Cytotoxicity Assay

The MTT (3-(4,5-dimethylthiazol-2-yl)-2,5-diphenyltetrazolium bromide) assay is a colorimetric assay to measure cellular metabolic activity as an indicator of cell viability, proliferation and cytotoxicity. J744A.1 cells were seeded in a 96-well plate with concentration 10^4^ cells/100μL/well and incubated for 24 h. After the medium was removed, 100 μL fresh medium containing peptide (ranging from 64 μg/mL to 2 μg/mL) was added to the wells. Following 24 h incubation, fresh medium with MTT (0.5 mg/mL) was replaced and incubated for 3 h. After the medium/MTT was removed, DMSO was added at 100 μL for dissolving the formazan crystal. Cell survival rate was calculated by measuring the absorbance at 540 nm using a Tecan Sunrise microplate reader (Tecan, Männedorf, Switzerland). Medium without peptide and mixed with H_2_O_2(aq)_ represented positive and negative controls, respectively.

### 4.6. Preparation of Large Unilamellar Vesicles (LUVs)

Large unilamellar vesicles (LUVs) of POPC: LPS (12.5:1, mol/mol), POPC: cholesterol (2:1, mol/mol) and POPC: POPG (3:1, mol/mol) were prepared by the extrusion method with an Avanti small-volume extrusion apparatus (Avanti Polar Lipids) as described elsewhere [13]. Extrusion procedure is the sequential passage of a dilute liposome preparation through polycarbonate filters of decreasing pore size, using a hand-held syringe and filter holder attachment, in order to produce a homogeneous size distribution [69,70]. Briefly, phospholipids were weighed and dissolved in chloroform: methanol (4:1, *v*/*v*) and then evaporated by nitrogen gas to form the lipid film. After suspending in PBS buffer, the lipid suspensions were frozen and thawed 6−8 times. Lipid suspensions were extruded with an extruder ten times through a 0.4 mm pore size polycarbonate filter (Avanti Polar Lipids), followed by ten times through a 0.1 mm filter.

Calcein-filled LUVs were prepared from calcein-containing buffer (70 mM calcein and 10 mM Tris at pH 7.4). Unencapsulated calcein was removed by gel filtration with a Sephadex G-75 column loaded with iso-osmotic buffer (100 mM sodium chloride and 10 mM Tris). The phospholipid content of vesicles was determined by assessing inorganic phosphate according to John Charles Marshall Stewart [71].

### 4.7. Dye Leakage Experiments

Peptide-induced calcein leakages were measured by Perkin–Elmer luminescence spectrofluorimeter at excitation and emission wavelengths of 496 and 515 nm, respectively. The concentration of calcein-entrapped LUVs was diluted to 10 μM. Leakage of 100% was induced by 100 mg/mL Triton X-100. The degree of leakage induced by various concentrations of peptides was estimated by: %leakage = ((F − F_0_)/(F_r_ − F_0_)) × 100%, where F_0_ and F_r_ are the initial fluorescence intensities observed without peptide and after addition of 100 mg/mL Triton X-100, respectively.

### 4.8. Circular Dichroism Spectroscopy

CD spectra were recorded with an AVIV 202 spectropolarimeter after calibration with d-l0-camphorsulfonic acid. All the measurements reported were carried out in 20 mM phosphate buffer and scanned wavelength from 190 to 260 nm at 25 °C in a 1 mm path-length cuvette. Three scans were averaged for each spectrum with a 0.2 nm step size. Peptide and liposome concentrations were diluted to 60 μM and 1 mM by using 20 mM phosphate buffer at pH 7.4. The appropriate baselines were used to subtract the data and the corrected data were converted to molar ellipticity (deg·cm^2^·dmol^−1^).

### 4.9. Dynamic Light Scattering

The LPS molecules were dissolved in chloroform: methanol (2:1) then the stock solution was sonicated at 40 °C for 20 min and then kept at 4 °C overnight. The stock solution was diluted to 25 μM by using 20 mM sodium phosphate buffer with 150 mM sodium chloride at pH 7.4 and then kept at 4 °C overnight before measurements. Peptide concentrations were diluted to 8 or 16 μg/mL. The particle size and distribution analysis was measured by dynamic light scattering measurements in Malvern Zetasizer ZS (Malvern, UK), equipped with a He-Ne laser. Three measurements (with 10 runs each) were taken by using disposable polystyrene cells at 37 °C.

### 4.10. Inhibition of Endotoxin-Induced Inflammatory

J744A.1 cells were seeded in a 24-well plate with 3 × 10^5^ cells per well and incubated for 24 h. Cells were washed by PBS and 300 μL phenol red-free DMEM medium containing lipopolysaccharides (LPS) from *Escherichia coli* 026:B6 (sigma Aldrich, 150 ng/mL) and peptide (ranging from 32 μg/mL to 2 μg/mL) were added to the wells. Untreated cells and LPS only treated cells served as positive and negative controls, respectively. Following 24 h incubation, culture supernatant was harvested and centrifuged at 1000 rpm for 10 min.

To investigate nitric oxide (NO) production, 50 μL culture supernatant was mixed with 50 μL Griess reagent (Sigma Aldrich, St. Louis, MO) in a 96-well plate and incubated at room temperature for 10 min. The absorbance at 540 nm was measured and the NO concentration was calculated by a standard curve generated with sodium nitrite (NaNO_2_). The medium only was used as blank control.

Concentrations of proinflammatory cytokine (TNF-α) in the culture supernatant were evaluated using mouse enzyme-linked immunosorbent assay kits for TNF-α (eBiosciences).

### 4.11. Endotoxemia Mouse Model

The animal model was described in our previous study [55]. Briefly, 5-week-old male C57BL/6 mice were purchased from the National Laboratory Animal Center (Taiwan). All animal experiments were performed in accordance with the animal guidelines of the National Tsing Hua University Institutional Animal Care and Use Committee. All experimental protocols were approved by the National Tsing Hua University Institutional Animal Care and Use Committee. All mice were sacrificed under CO_2_, and all efforts were made to minimize suffering. The weight of each mouse was approximately 22.5 g at the start of the experiments. Mice were divided into three groups (5 in each group with intraperitoneal (i.p.) injection of 18 mg/kg of body weight *Escherichia coli* O26:B6 LPS alone or 18 mg/kg LPS plus 10 mg/kg peptides or no treatment control). Blood was collected via tail vein 1.5 h after injection. Whole blood was centrifuged at 3000 rpm at 4 °C for 10 min, and supernatant was collected and measured by mouse TNF-α enzyme-linked immunosorbent assay (ELISA) kits (eBioscience). After 24 h, all mice were sacrificed. The lungs were removed and fixed in 4% formaldehyde buffer. Paraffin-embedded tissues were cut into 2 μm-thickness sections, and deparaffinized in ultraclear buffer (J.T. Baker) and graded ethanol. The morphology of the lungs was obtained by H&E stained sections. Tissue images were captured using light microscope (ECLIPSE TE2000-E, Nikon) with a camera (D50, Nikon) at 40X fields.

### 4.12. Statistical Analysis

All in vitro results are presented as means ± SD. Levels of significance were calculated using one-way ANOVA, followed by student’s t-tests, using GraphPad Prism software (significance between data with a threshold of * *p*  <  0.05; ** *p* < 0.01; *** *p* <  0.001).

## Figures and Tables

**Table 1:** Primary structure, charge, hydrophobicity and molecular weight of Ala-Gly-Pro (AGP) series peptides.

Peptide	Sequence	Charge	Hydrophobicity <H>	Molecular Weight (Da)
KR12	Ac-KRIVQRIKDFLR-NH_2_	+4	0.193	1517.93
RW6	Ac-RRWWRW-NH_2_	+3	0.62	1045.22
KR12AGPKR6	Ac-KRIVQRIKDFLR-AGP-IKDFLR-NH_2_	+4 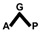 +3	0.193 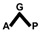 0.62	2611.2
KR12AGPWR6	Ac-KRIVQRIKDFLR-AGP-RRWWRW-NH_2_	+4 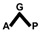 +3	0.193 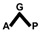 0.282	2865.46
KR12AGPVR6	Ac-KRIVQRIKDFLR-AGP-RRLVRI-NH_2_	+4 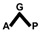 +3	0.193 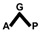 0.282	2632.27

The physicochemical parameters were calculated on the website: http://heliquest.ipmc.cnrs.fr/.

## Abbreviations

AMPs	antimicrobial peptides
ATCC	American Type Culture Collection
BCRC	Bioresource Collection and Research Center
Bip	β-(4,40-biphenyl)alanine
CD	circular dichroism
CFU	colony forming unit
CSE	control standard endotoxin
Dip	β-diphenylalanine
DLS	dynamic light scattering
DMEM	Dulbecco’s modified eagle’s medium
DMSO	dimethyl sulfoxide
ELISA	enzyme-linked immunosorbent assay
EU	endotoxin unit
H&E	hematoxylin and eosin
HPLC	high performance liquid chromatography
i.p.	intraperitoneal
LAL	*Limulus* amebocyte Lysate
LPS	lipopolysaccharide
LUV	large unilamellar vesicles
MH broth	Mueller−Hinton broth
MIC	minimal inhibitory concentration
MALDI-TOF	matrix-assisted laser desorption-ionization time-of-flight
MTT	3-(4,5-dimethyl-2-thiazolyl)-2,5-diphenyl-2H-tetrazolium bromide
Nal	β-naphthylalanine
NF-κB	nuclear factor-kappa B
NO	nitrite oxide
PBS	phosphate-buffered saline
POPC	1-palmitoyl-2-oleoyl-sn-glycero-3-phosphocholine
POPG	1-palmitoyl-2-oleoyl-sn-glycero-3-phospho-(10-rac-glycerol)
SD	standard deviation
TLR4	Toll-like receptor 4
TNF-α	tumor necrosis factor-alpha
